# Presence of Serum Ferritin before and after Bariatric Surgery: Analysis in Dentate and Edentulous Patients

**DOI:** 10.1371/journal.pone.0164084

**Published:** 2016-10-03

**Authors:** Gerson Aparecido Foratori, Francisco Juliherme Pires de Andrade, Victor Mosquim, Matheus de Carvalho Sales Peres, Reginaldo Ceneviva, Elinton Adami Chaim, Silvia Helena de Carvalho Sales Peres

**Affiliations:** 1 Department of Pediatric Dentistry, Orthodontics and Public Health, Bauru School of Dentistry, University of São Paulo, Bauru, São Paulo, Brazil; 2 Department of General Surgery, Botucatu School of Medicine, University of State of São Paulo, Botucatu, São Paulo, Brazil; 3 Department of Surgery and Anatomy, School of Medicine of Ribeirão Preto, University of São Paulo, Ribeirão Preto, São Paulo, Brazil; 4 Department of Surgery, Faculty of Medical Science of Campinas, State University of Campinas, Campinas, São Paulo, Brazil; University of North Carolina at Chapel Hill, UNITED STATES

## Abstract

Society has changed its own lifestyle, specially its eating habits and physical activities, leading to excessive weight and a sedentary behavior, which has contributed to obesity increase. Bariatric surgery is the most effective treatment to obesity, allowing weight loss and its maintenance. However, it has been related high levels of iron deficiency after surgery. A person’s nutritional status might be affected by total or partial tooth loss. The aim of this longitudinal prospective cohort study was to evaluate the levels of serum ferritin before and after bariatric surgery and to identify if there is a relation with tooth loss. The sample was composed of 50 patients selected and assisted at Amaral Carvalho Hospital, located in Jaú city, Brazil. The use and necessity of prosthesis, dental absence or presence, and serum ferritin dosage were evaluated. Student’s t test, Univariate analysis, Chi-square and Odds Ratio were adopted (p<0.05). There was no significant difference regarding the serum ferritin levels between dentate and edentulous patients prior to surgery (p = 0.436). After surgery, the serum ferritin levels were higher in edentulous patients (prosthesis users) when compared to the pre-surgical levels, and the post-surgical levels presented significant difference regarding the dentate patients (p = 0.024). It can be concluded that rehabilitated patients in postoperative period showed better levels of serum ferritin after surgical intervention.

## Introduction

The change in eating patterns, characterized by a high-calorie diet and reduction of complex carbohydrate and fiber intake, combined with a sedentary lifestyle have been comprehended as a key factor to obesity increase.

Obesity is a chronic disease that has reached pandemic proportions. This condition has been recognized as one of the main public health challenges [1], being related to important socioeconomic problems [2]. The cause of obesity is recognized by the association of metabolic, behavioral, genetic, social and cultural factors [3,4]. According to researches performed in the United States between 2013 and 2014, 70.7% of adults aged 20 or more are overweight, and 37.9% of that same group is obese [5]. In Brazil, according to Vigitel there was an increase in the number of people with excessive weight compared to 2006: 52.5% of Brazilian are overweight, whilst this percentage was 43% in 2006 and, still, 17.9% of the population is obese [6].

Bariatric surgery is the surgical treatment for obesity, which in turn reduces weight and to maintain its loss by restricting the intake and/or malabsorption of food [7]. This intervention has been proposed to patients with Body Mass Index (BMI) higher than 40kg/m^2^ or higher than 35kg/m^2^ associated to comorbidities, such as hypertension, dyslipidemia, type II diabetes, obstructive sleep apnea (OSA), among others. Various authors have reported low rates of iron after bariatric surgery [8,9].

The oral condition is strongly associated with obesity. Literature has shown that oral problems, such as dental caries, periodontal disease, change of the salivary flow volume, and mainly the tooth loss are etiological factors of obesity, since it contributes to weight gain process, including the period after surgery [10].

On the other hand, tooth loss harms the nutritional conditions, as it negatively interferes on the masticatory function, complicating the intake of nutritive food [11,12]. The poor supply of nutrients may cause damage to individuals by promoting systemic diseases and other alterations. Nutritional deficiency is one of the several consequences of edentulism, and it may be related to decreased masticatory function [13]. The wide teeth contact area allows an adequate food degradation. When teeth are absent, the rehabilitation with prosthesis may enhance the masticatory condition. Yet, nothing is as good as natural teeth [14].

Among the iron ion availability markers, serum ferritin is one of the most used markers, especially in epidemiological studies, and accurately reflects the body iron reserves [15]. Ferritin levels reflect the cell storage of iron. However, they may be related to inflammatory conditions. Obesity, once it shows a chronic inflammatory process, can also lead to an increase in the levels of ferritin. Several obese patients have iron deficiency possibly due to the sum of an inadequate diet (high-calorie and unbalanced) with a demand increase, owing to body length.

There are no studies involving obesity, serum ferritin levels and oral conditions. This fact highlights the need to seek if there is an association between the levels of serum ferritin with oral conditions before and after bariatric surgery. Therefore, this study contributes to scientific literature regarding the full health care of morbid obese patients.

Considering that edentulism adversely affects the nutritional status of patients, and also that both morbid obese and bariatric patients may have serious oral consequences, this present study aimed to evaluate the serum ferritin levels before and after bariatric surgery in order to identify the relation with tooth loss in these patients.

## Material and Methods

The STROBE guidelines were used to ensure the reporting of this observational longitudinal prospective cohort study [16]

### Ethical aspect

In respect to the Declaration of Helsinki guidelines, this study was submitted to and approved by the Ethics Committee on Human Research of the Bauru School of Dentistry, University of São Paulo (process 014/2011) and Ethics Committee on Human Research of the Amaral Carvalho Foundation (process 111/2011). All subjects signed a written consent form regarding their participation in the study.

### Anthropometric assessment

The Body Mass Index (BMI) was obtained by the patient’s weight (kg) divided by their height squared (m^2^). Weight was obtained in an automatic scale (MIC model 300PP, Micheletti Ind., max capacity 300kg) and height in a stadiometer (Wood 2.20, WCS Ind., Brazil). Subjects were classified as morbid obese when BMI≥40.0kg/m^2^. The surgery was only an option to patients with BMI higher than 40kg/m^2^ or higher than 35kg/m^2^ associated to comorbidities.

### Sample composition

This is an observational longitudinal prospective cohort study. The team of dentists of Bauru School of Dentistry assisted obese patients intending to undergo bariatric surgery at the clinic of Amaral Carvalho Hospital (Jaú/SP) before and after the intervention. All necessities of oral conditions were treated before bariatric surgery. The patients with tooth loss were only allowed to undergo surgery after prosthetic rehabilitation with total and/or removable partial prosthesis. Sample size was calculated by using Statistica software (StatSoft Inc., Tulsa, USA), considering significance level set at 5%, 80% of power, and a sample ratio of 2:1. A pilot study was conducted to verify the proposed strategies, testing its applicability and previous results were analyzed. This pilot study consisted of a ten-patient sample submitted to bariatric surgery.

**Fig 1:**
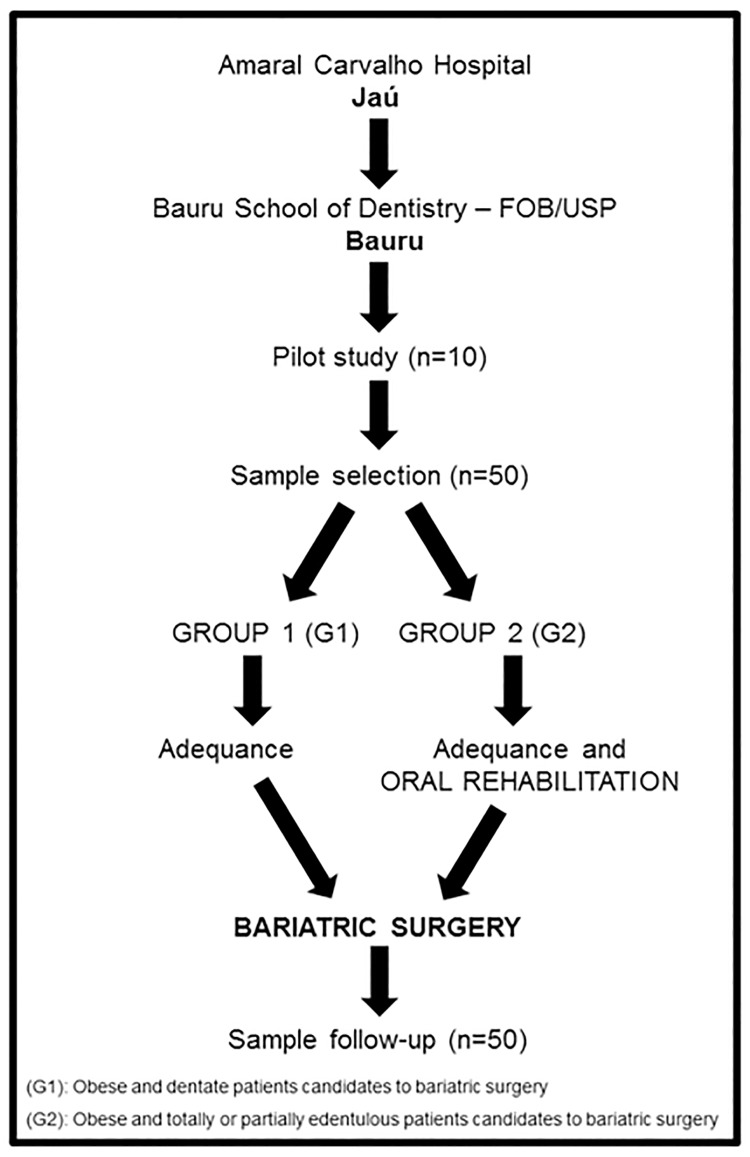
Study design.

According to the pilot study, the applicability analysis was performed. Then, the sample consisted of 50 patients assisted at the clinic of Amaral Carvalho Hospital (Fig 1). The eligibility criteria selected patients, who had to be under prior regular medical attendance and with stable systemic health condition, even if presented any comorbidities. Adopted exclusion criteria dismissed patients who did not present the minimum period of three months after surgery, not registering their serum ferritin levels post-operatively. Additionally, the selected patients must present satisfying oral conditions, which were adequate by the dental staff of Bauru School of Dentistry prior to bariatric surgery.

### Serum ferritin levels

Serum ferritin levels were obtained before and after surgery from medical records of Amaral Carvalho Hospital. Post-operatively the time spent since the surgery and the amount of weight loss in excess were registered. In order to measure the levels of serum ferritin, the values proposed by the International Nutritional Anemia Consultative Group were adopted (Table 1) [17].

**Table 1:** Used criteria to characterize the serum ferritin levels.

Classification	Ferritin (ng/mL)
Deficient	< 12.0
Doubtful	12.0–20.0
Adequate	> 20.0

### Oral Examination

Oral examinations were conducted by a previously calibrated dentist (Kappa>0.88). A plain oral mirror, a clinical probe n. 05 and a syringe with compressed air were used to exam the oral cavity. In the pre-operative period, the oral conditions related to the presence or absence of teeth, the use and need of prosthesis and the type of prosthesis used were registered on a previously elaborated medical record.

Regarding oral health, the data followed the proposals of World Health Organization (WHO) for the use (do not use dental prosthesis, uses a one fixed partial prosthesis; uses more than one fixed partial prosthesis; uses removable partial prosthesis; uses one or more fixed partial prosthesis with one or more removable partial prosthesis; uses total prosthesis; no information) and need (do not need prosthesis; needs a fixed or removable partial prosthesis for one element replacement; needs a fixed or removable partial prosthesis for more than one element replacement; needs a combination of prosthesis, fixed and/or removable, for the replacement of one and/or more than one element; needs a total prosthesis; no information) of prosthesis. In addition, the presence or absence of teeth was noted, where the presence of at least 20 teeth in function was relevant.

Oral health maintenance was evaluated in both dentate and edentulous patients during the post-operative period. Furthermore, prosthesis of the edentulous patients were analyzed to make sure there still were in appropriate conditions.

### Statistical analysis

Data were organized with double entry in files of Excel for Mac 2011. After, descriptive analysis (mean, standard deviation, absolute and relative frequencies) was performed. Regarding the statistical analysis, *Statistica 10*.*0* for Windows was used. Initially, homogeneity, equal variance and normal distribution were tested by Kolmogorov-Smirnov and Shapiro-Wilk. In order to evaluate the difference between pre- and post-operative periods in both groups, Student’s t test was applied. Univariate analysis, Chi-square and Odds Ratio were adopted to analyze the serum ferritin levels before and after bariatric surgery. Significance level of 5% was admitted.

## Results

This study was composed of ten pilot and fifty morbid obese patients. The total number of individuals in the sample (n = 50) was divided into two groups. The edentulous group (total or partial edentulous), composed of 21 subjects (42% of the sample), with 17 women and 04 men, and among the dentate group, which was composed of 29 subjects (58% of the sample), there were 18 women and 11 men (Table 2).

**Table 2:** Distribution of the sample according to oral health

Oral conditions	n	% of individuals
Dentate	29	58
Edentulous	21	42

Within the edentulous group (total or partial edentulous), the teeth that had been most commonly absent were analyzed (Table 3).

**Table 3:** Missing teeth in edentulous patients.

Absent teeth	% of individuals with tooth loss
Incisors	40
Premolars	88
Molars	100

All edentulous patients were prosthetically rehabilitated with total dental prosthesis and/or removable partial prosthesis during the preoperative period (Table 4).

**Table 4:** Distribution of patients using dental prosthesis.

Sample	n	% of users of dental prosthesis in the postoperative period
Dentate	29	0
Edentulous	21	100

In the preoperative period, there was no significant difference in the levels of serum ferritin between dentate and edentulous patients (Table 5).

**Table 5:** Univariate analysis of serum ferritin levels preoperatively according to the condition of present and absent teeth.

Oral Conditions	Serum Ferritin				
	Altered	Normal	Total	Odds-Ratio	X^2^ (p)
Edentulous	13	8	21	CI (95%) /0.444 (0.056–3.508)	0.61 (p = 0.436)
Dentate	24	5	29		
Total	37	13	50		

After bariatric surgery, there was an increase regarding serum ferritin levels in edentulous individuals, yet denture users (total dental prosthesis and/or removable partial prosthesis). This result presented a significant difference compared to the group of dentate patients (Table 6).

**Table 6:** Univariate analysis of serum ferritin levels postoperatively according to the condition of present and absent teeth.

Oral Conditions	Serum Ferritin				
	Altered	Normal	Total	Odds-Ratio	X^2^ (p)
Dental prosthesis	5	16	21	CI (95%) /0.109 (0.109–0.871)	5.07 (p = 0.024)^a^
Dentate	24	5	29		
Total	29	21	50		

^a^ Significant difference.

The average weight loss after 3 months of the intervention was 33% for the group of dentate and 31% for the group of edentulous that were rehabilitated with total dental prosthesis and/or removable partial prosthesis (Table 7).

**Table 7:** Comparison of loss percentage of excess weight after 3 months of surgery between dentate and rehabilitated patients.

Oral Conditions	n	Loss of excess weight after 3 months of surgery (%)
Dentate	29	33
Dental prosthesis	21	31

## Discussion

This research sought to contribute to literature by investigating the association among obesity, bariatric surgery, tooth loss and serum ferritin levels. The results evidenced that the levels of serum ferritin became increased in prosthetically rehabilitated patients after bariatric surgery compared to preoperative period, when they were not prosthesis users yet.

Obesity growth in developed and in developing countries has been considered alarming, being one of the most neglected contemporary health problems around the globe [18]. A longitudinal study from 1980–2013 compared the prevalence of overweigh and obesity worldwide. The results of the cited study showed an increase in the proportion of male adults with a BMI equal to or higher than 25 kg/m^2^, growing from 28.8% in 1980 to 36.9% in 2013. The number of female adults with a BMI equal to or higher than 25 kg/m^2^ enlarged from 29.8% to 38% during the same period. In 2010, overweight and obesity were the cause of 3.4 million deaths around the world [19].

In this present study, the sample consisted mostly of women (73.3%), although there is no difference regarding the obesity’s distribution by gender in Brazil [20]. These findings corroborate to the studies of Harris and Barger, which 83% of the patients undergoing bariatric surgery were female [21]. Psychologically, women are more affected by overweight, once the ideal female body is related to a thin figure as a symbol of attractiveness, beauty and success [22].

Different surveys have reported obesity related oral problems, such as dental caries, periodontal disease and bone loss. Even though the studies relating dental caries and obesity are controversial, several researches suggest that obese patients are more susceptible to dental caries [3,23,24].

It is well established in the literature that overweight and obesity are related to periodontitis, since obesity inflammatory mediators may interfere in the periodontal disease susceptible host [25,26].

Haffajee and Socransky evidenced one possible explanation to the link between obesity and periodontal disease. They found *Tannarella forsythia* in the gingival fluid of obese individuals, who present a higher risk to developing periodontitis [26,27]. In a certain study of Sales-Peres et al, it was established *Porphyromonas gingivalis*, *Tannarella forsythia*, *Treponema denticola*, and *Prevotella intermedia* were found in 71%, 80%, 72%, and 85% of the patients before surgery, respectively [28]. The relationship between obesity and periodontal disease is extremely important for public health, considering that both are risk factors for cardiovascular diseases [26,28–31].

Other findings have linked obesity with bone loss [10,32,33]. It has been suggested that obese patients with bone loss may present missing teeth, which could damage their masticatory function. Even though tooth loss etiology was not assessed in this study, it is defined in literature that dental caries and periodontitis are the main causes for tooth loss [34].

Regarding the sample of this study, 42% of patients were totally or partially edentulous and 58% were dentate (Table 2). This result confirms that almost half of patients that seek treatment for obesity in the Public Health System in Brazil need dental treatment before surgical intervention.

Concerning the tooth loss, all partially or totally edentulous patients had molar loss, and 80% of these patients had lost premolars pre-surgically (Table 3). It reduces masticatory efficiency, harming the nutrition and, consequently, the surgery outcome of these patients.

Excessive weight negatively influences the quality of life of individuals. Bariatric surgery is the surgical treatment for obesity. Mostly, this surgery has satisfying results concerning weight loss and its maintenance, assuring better quality of life to these individuals [10,18,35–39]. However, this treatment may also have some negative consequences, such as hyperparathyroidism, osteoporosis [10,40–42], chronic regurgitation [10,43], nutritional deficiencies [10,40,44–46], kidney problems [10,47,48] and oral problems [10,28,49–51].

The bariatric surgery is recommended for morbidly obese individuals, promoting weight reduction and remission of comorbidities, for example: diabetes, cardiovascular complications and mortality [52–57]. Roux-en-Y gastric bypass (RYGB) has been cited as a type of bariatric surgery with 85% of patients receiving this restrictive/malabsorptive procedure [57–59]. Considering that in this technique gastric capacity is reduced by 90–95%, adverse effects such as nutritional deficiencies and gastrointestinal symptoms have been observed, including disturbances of anatomical and physiological functions [57,60]. All patients in this study were submitted to Roux-en-Y gastric bypass.

Some limitations can be found in this study, once factors like regurgitation and osteoporosis, that may cause dental erosion and alveolar bone loss, respectively, could not be investigated. Considering that gastroesophageal reflux and alterations in the levels of vitamin D and calcium might occur after surgery, future studies ought to be conducted in order to establish a relation between these variables.

The association between bariatric surgery and periodontal disease is well evidenced in the scientific scenario [28,51,61]. One explanation to the worsening of the periodontal status after the surgical intervention for obesity is based on the nutritional deficiency these patients have after surgery. The lack of vitamin D, for example, may cause metabolic bone deficiency, deteriorating the periodontal condition of these patients [28,62,63], being a predisposing factor for tooth loss.

Bariatric surgery demands new eating habits, such as ingesting doughy food, which must be chewed properly. Therefore, the subject starts eating a small amount of food, yet more frequently, demanding good oral hygiene practice.

The association between oral problems and bariatric surgery requires the inclusion of dentist in the multidisciplinary team of care for morbid obese patients, candidates for this treatment, in order to providing complete care, guaranteeing satisfying outcomes after bariatric surgery.

In Brazil, about 70% of population have missing teeth, indicating the need to rehabilitation using total or partial prosthesis (fixed and/or removable) [64]. Obese patients and candidates to bariatric surgery show systemic particularities, which is commonly characterized by the nutritional deficiency. In these situations, the tooth loss becomes another predisposing factor to the nutritional problem imposed by deficient mastication.

All edentulous patients were prosthetically rehabilitated using total and/or partially removable prosthesis before undergoing bariatric surgery (Table 4).

In general, nutritional problems, such as anemia, osteoporosis and metabolic disease [10,65] affect approximately 30% of patients who have undergone bariatric surgery. The most common problems are anemia and deficiency in the levels of iron, which represent 54.4% and 36.6% of cases, respectively [10]. In the few published studies, it was established that a significant part of morbid obese patients suffer from nutritional deficiencies even before surgery [66], and after surgery, it may reach up clinical and subclinical proportions [67].

Anemia is the result of a wide variety of causes that often coexist with other micronutrient deficiencies. Iron deficiency is the most significant contributor to anemia [68–70]. Furthermore, studies show maternal education, gender norms, and low income as different causes to anemia related to socioeconomic factors [70,71]. Infectious diseases, such as malaria, worm infestation and schistosomiasis, are also associated to anemia, [70,72–74] and deficiencies of other essential micronutrients such as vitamin A, folic acid and vitamin B12 as well [70,75]. World Health Organization estimated that about 40% of the world’s population (more than 2 billion people) suffer from anemia [70,76]. This condition impairs the immune mechanisms, and causes increased morbidity which may lead to fatigue, low productivity, and a general sense of feeling unwell [70,77].

It is important to highlight that obesity is a known risk factor for nutrient deficiencies. Some studies even informed that a significant proportion of patients already had anemia prior to RYBG surgery [57,78–80]. Yet, a wide range of studies has reported an increase in the prevalence and severity of anemia after RYGB surgery, but the causes are not fully understood [45,57,81–83].

According to a study conducted by Bavaresco et al, the iron deficiency can be found in 12.2% of obese patients pre-surgically and 14.6% one year after the operation [84]. Another study detected iron lack in 40% of patients two years after surgery and in 54% after three years from the surgery [85].

Ferritin is an intracellular protein responsible for fixing, storage and releasing of iron. Consequently, it is the main protein involved in the regulation and availability of iron ion. The measurement of ferritin in plasma is widely used to evaluate this ion concentration in the organism with the intention of analyzing its privation or overload [86].

In adults, the lack of iron tends to cause ineffective hematopoiesis, leading to anemia, reduction of the work capacity and damaged immune system [87–89]. In contrast, the high iron stores are associated with an increased risk of diabetes mellitus, atherosclerosis and cancer. Therefore, a proper immune system and nutritional balance, serum ferritin levels must be within the normality range [90–92].

Considering gastric bypass of RYGB has dual effects on intake restriction and malabsorption, some studies had showed iron deficiency in the patients after the procedure [57,93]. Many conditions can cause anemia after RYGB surgery, such as patients’ caloric intake reduction with concomitant changes in eating behavior and dietary adherence; hypochlorhydria, the virtual absence of hydrochloric acid secretion by the stomach; and last of all, ingested food bypasses through most of the stomach and duodenum, as well as a significant portion of the jejunum, where physiological iron absorption takes place [57,81,94–97]. Literature shows that patients who do not respond to oral supplementation should be referred early on for parenteral replacement therapy to prevent anemia [57,98].

At baseline, both obese dentate patients and obese edentulous patients had a poor dietary intake. This type of feeding is called black and white diet, because these patients prefer foods like rice, beans, bread, coffee and toasts over colorful vegetables, which are really nutritive. Furthermore, these patients consume small amounts of meat that is rich in nutrients. The only colorful food that these patients consumed were colored artificially, which contributed to weight gain. These food patterns could partly explain the low probabilities of micronutrients adequacy of our patients, thus increasing their risk of nutritional deficiencies. Although obese dentate group were able to eat more meat when compared to the other group, due to their more efficient chewing, both presented low serum ferritin levels before the bariatric surgery.

The results of this research revealed that during the pre-surgical period, dentate and partially or totally edentulous patients presented altered levels of ferritin (Table 5). Thus, there was no statistical difference between both groups (p = 0.436). However, during the post-surgical period, the levels of ferritin in the prosthetically rehabilitated patients increased when compared to the pre-surgical levels. In this period, these rehabilitated patient were classified in the normality range due to presenting higher serum ferritin levels when compared to the pre-surgical ferritin concentration (Table 6), showing a significant difference in contrast to the dentate group (p = 0.024). Some findings have exposed that among the causes commonly related to iron deficiency are food intolerance, gastric acid secretion and decreased absorption due to duodenum exclusion [99].

According to the data of this study, it was expected that the preoperative levels of serum ferritin were altered due to the nutritional deficiency presented by all patients, once obesity causes a general chronic inflammation associated to an inadequate food intake.

Moreover, post-operatively it was expected that these patients kept altered serum ferritin levels, as the reduction of the gastroesophageal tract was executed. All patients of this study had nutritional attendance before and after bariatric surgery. However, after the procedure, all of them were likely to develop altered frequency and quantities patterns of feeding and deficient absorption due to the surgery performed. This fact is correlated with the study conducted by Aron-Wisnewsky et al that observed suboptimal food intakes after gastric bypass, leading to a risk of micronutrient deficiencies [100].

Nevertheless, it is important to highlight that, after 3 months of the surgical treatment, even with difficulties, all the patients started to prioritize colorful foods that are really rich in nutrients such as fruits and vegetables. They started to give preference for protein, iron and calcium sources, especially those patients who were rehabilitated before the bariatric surgery, once they had an improvement of the masticatory function.

In this study only the dentate group preserved altered levels of ferritin. The group of edentulous patients, who had recently been rehabilitated with dental prosthesis, began to show normal levels of serum ferritin. This occurrence may be explained by the improvement in masticatory function of these patients. Consequently, they started to chew meat, fish and nutritive food that were not likely to be chewed before. Even with the reduction of the gastroesophageal tract, the prosthesis users started to chew better, resulting in an increase of ferritin levels.

The prosthetic rehabilitation of these patients started to contribute to food digestion and nutritive food choice, improving the nutritional status in general, especially as concerns the serum ferritin levels. Some studies evidenced that proper chewing enhance the initial steps of digestion due to the stimulation of saliva production and activation of cephalic controls, which initiate food assimilation function [101].

After 90 days of the surgery intervention, the percentage of excess weight loss was similar in both groups (Table 7). The mean weight loss after 3 months in the dentate group individuals was 33%, and for the group of prosthetically rehabilitated edentulous patients was 31%. This finding leads to reflection about the importance of the use of prosthesis, which in turn affects the nutritional status. Although well adapted total and/or partial removable prosthesis improves the totally or partially edentulous patients’ condition, it does not have similar performance to natural dentition regarding food trituration [102–105].

Literature points out that prosthesis users have diets with lack of fiber and vitamins, due to the difficulty in chewing certain foods [106].

Scientific evidence shows that maximum biting force in total prosthesis rehabilitated patients is 4.5 times lower than in patients with natural healthy dentition [107]. Other studies found the average number of 4 kgf for chewing force with total prosthesis [108]. Concerning natural teeth, it was registered a value of 9 kgf for chewing force [109]. In the studies conducted by Widmark et al, on the contrary, the value of 36 kgf for biting force and 12.5 kgf for chewing force were recorded regarding natural teeth [110]. Therefore, the interaction among dental prosthesis effectiveness, weight loss and nutrition status in patients who underwent bariatric surgery must be established by future longitudinal studies.

## Conclusion

After at least 90 days from the bariatric surgery, the prosthetically rehabilitated individuals presented better serum ferritin levels than dentate patients. This result showed an improvement of these levels when compared the preoperative and postoperative periods in these patients.

These findings highlight that morbid obese patients who were candidates to bariatric surgery as well as patients that have already undergone this surgical procedure require follow-ups with dentists in order to be fully treated. It may improve not only the results of this kind of surgery, but also the quality of life of these patients.
